# Enhanced trypsin on a budget: Stabilization, purification and high-temperature application of inexpensive commercial trypsin for proteomics applications

**DOI:** 10.1371/journal.pone.0218374

**Published:** 2019-06-27

**Authors:** Søren Heissel, Sigurd J. Frederiksen, Jakob Bunkenborg, Peter Højrup

**Affiliations:** 1 Department of Biochemistry and Molecular Biology, University of Southern Denmark, Odense M, Denmark; 2 Alphalyse A/S, Odense SØ, Denmark; Shantou University Medical College, CHINA

## Abstract

Trypsin is by far the most commonly used protease in proteomics. Even though the amount of protease used in each experiment is very small, digestion of large amounts of protein prior to enrichment can be rather costly. The price of commercial trypsin is highly dependent on the quality of the enzyme, which is determined by its purity, activity, and chemical modifications. In this study we evaluated several strategies for improving the quality of crude trypsin by reductive methylation and affinity purification. We present a protocol applicable to most proteomics laboratories for obtaining a highly stable and pure trypsin preparation using reductive methylation and purification by benzamidine-sepharose. The entire workflow can be performed within a day and yields ~4 mg per batch but is completely scalable. The methylated product was benchmarked against sequencing grade trypsin from Promega and they were found to be comparable for one hour digestions at elevated temperatures, where residual chymotryptic activity was found to be negligible.

## Introduction

Trypsin is a serine protease produced in the pancreas of many vertebrates. It is initially produced as the inactive zymogen trypsinogen, but it becomes enzymatically active upon proteolytic cleavage in the small intestine [1]. Active trypsin is a highly specific protease that hydrolyzes peptide bonds C-terminal to lysine and arginine while having decreased efficiency when these are flanked by acidic residues or followed by proline (known as the Keil rules[2]) and also low efficiency towards terminal cleavage positions.

Proteolytic cleavage by trypsin primarily results in peptides with a length of 8–15 residues, which is optimal for RP-HPLC and the current generation of mass spectrometers. This generation of basic C-terminals leads to strong y-ion series when fragmented by collision-induced dissociation[3] (CID) and improves peptide identification in MS/MS-experiments. For these reasons, in combination with the fact that crude trypsin is relatively cheap and can be obtained in relatively high purity, trypsin has become the most widely used protease in bottom-up proteomics workflows[4].

The trypsin:substrate ratio commonly used is 1:20–1:100 w/w, and with increasingly sensitive instruments, the consumption of trypsin can be rather low. However, the analysis of low-stoichiometric PTMs or low-abundant protein contaminants, such as host-cell proteins (HCPs), may require digestion of vast amounts of starting material, thus requiring large amounts of trypsin, which induces a significant cost[5].

Commercial trypsin is available from many vendors and in many different forms. Most trypsin preparations contain minor non-tryptic activity from co-purified chymotrypsin, unless a recombinant[6] or chemically modified version of trypsin [7] is used. The most expensive trypsin preparations are very pure with negligible to no detectable trace activity from contaminating proteases and have furthermore been chemically modified in order to increase the specificity and/or stability of the protease.

During protein digestion, trypsin will hydrolyze itself, forming a nicked version named pseudotrypsin, which has been reported to display chymotryptic activity[8]. Tryptic autodigestion will furthermore decrease the proteolytic activity over time while increasing the complexity of the sample matrix by introducing various autocleavage products. Decreasing autodigestion of trypsin by modification of the basic residues has been reported on numerous occasions through the 1950’s to 1970’s with varying effectivity[9–12]. A common stabilizing modification is reductive methylation of primary amines using formaldehyde, which converts lysine into dimethyllysine whereby it is no longer is a suitable substrate for trypsin[13]. The surge of proteomics has resulted in an increase in the use of trypsin, and the augmented stability of modified trypsin has been shown to enable fast tryptic digestions at elevated temperatures[14–16].

Several protocols for reductive methylation use sodium cyanoborohydride as a reducing agent. However, due to its high toxicity, it is desirable to use a less toxic chemical, such as 2-picolone borane complex, which has been reported to an effective substitute[17]. An important factor to consider, when carrying out methylation, is that the reaction buffer is free of primary amines. Several protocols for dimethyl labeling of peptides suggest the use of triethylammonium bicarbonate (TEAB) at pH ~8.5. However, as this pH coincides with the pH optimum of trypsin, it may cause excessive autodigestion and reduce the yield. A study in 2013 reinvestigated the optimal pH for dimethyl labeling of peptides and found that pH 5–6 resulted in more extensive labeling[18].

In this study we evaluated several strategies for increasing the stability, purity and specificity of crude porcine trypsin in order to make a cheap, fast and effective protocol for proteomics laboratories. The work can easily be replicated and will help reduce the cost of protease when performing proteomics experiments.

To stabilizef trypsin we carried out reductive dimethylation of lysine residues using formaldehyde. We evaluated the extent of dimethylation at different pH values, incubation times and using either sodium cyanoborohydride or 2-picoline borane complex as reducing agents. The purification of trypsin was carried out using benzamidine-sepharose beads, which has previously been determined to be an efficient purification strategy for trypsin and it also has the advantage of increasing the activity of trypsin preparations[19]. Furthermore, the affinity of pseudotrypsin for benzamidine has been reported to be lower than that of trypsin[20].

We evaluated the loading of trypsin onto the benzamidine-sepharose beads in high-salt buffer and saturated L-phenylalanine buffer. A high-salt concentration should reduce non-specific binding and thereby reduce the amount of co-purified contaminating proteases. Loading trypsin in a saturated L-phenylalanine solution might decrease chymotrypsin’s affinity for benzamidine, allowing it to be removed during washing. In order to elute trypsin from the sepharose beads we evaluated the use of 12 mM HCl and varying concentrations of L-arginine. The low pH of HCl should cause trypsin to dissociate from the beads, while a high concentration of L-arginine should function as a competitor to benzamidine and therefore promote elution of trypsin, while chymotrypsin remains bound to the beads[21].

The specificity and activity of the methylated and benzamidine-purified trypsin (henceforth referred to as enhanced trypsin) was characterized and benchmarked against Promega sequencing grade trypsin (henceforth referred to as PSG), as it represents one of the highest quality trypsins available[5]. The entire protocol can be performed in a few hours and with minimal cost in terms of chemicals. The obtained product was determined to be very pure and highly stable towards autodigestion and at the same time significantly cheaper than commercially available methylated trypsin. The starting material in this study, porcine trypsin type IX-S from Sigma-Aldrich, was found to be a very pure and efficient protease already, but was also found to be contaminated by some chymotryptic activity, which was not completely removed by methylation alone.

## Material and methods

### Materials and data repository

All chemicals and reagents were purchased from Sigma-Aldrich, Germany, unless otherwise stated.

The mass spectrometry proteomics data has been deposited in the ProteomeXchange Consortium via the PRIDE[22] partner repository with the dataset identifiers PXD008193 and PXD013458.

### Protocol

**Fig 1:**
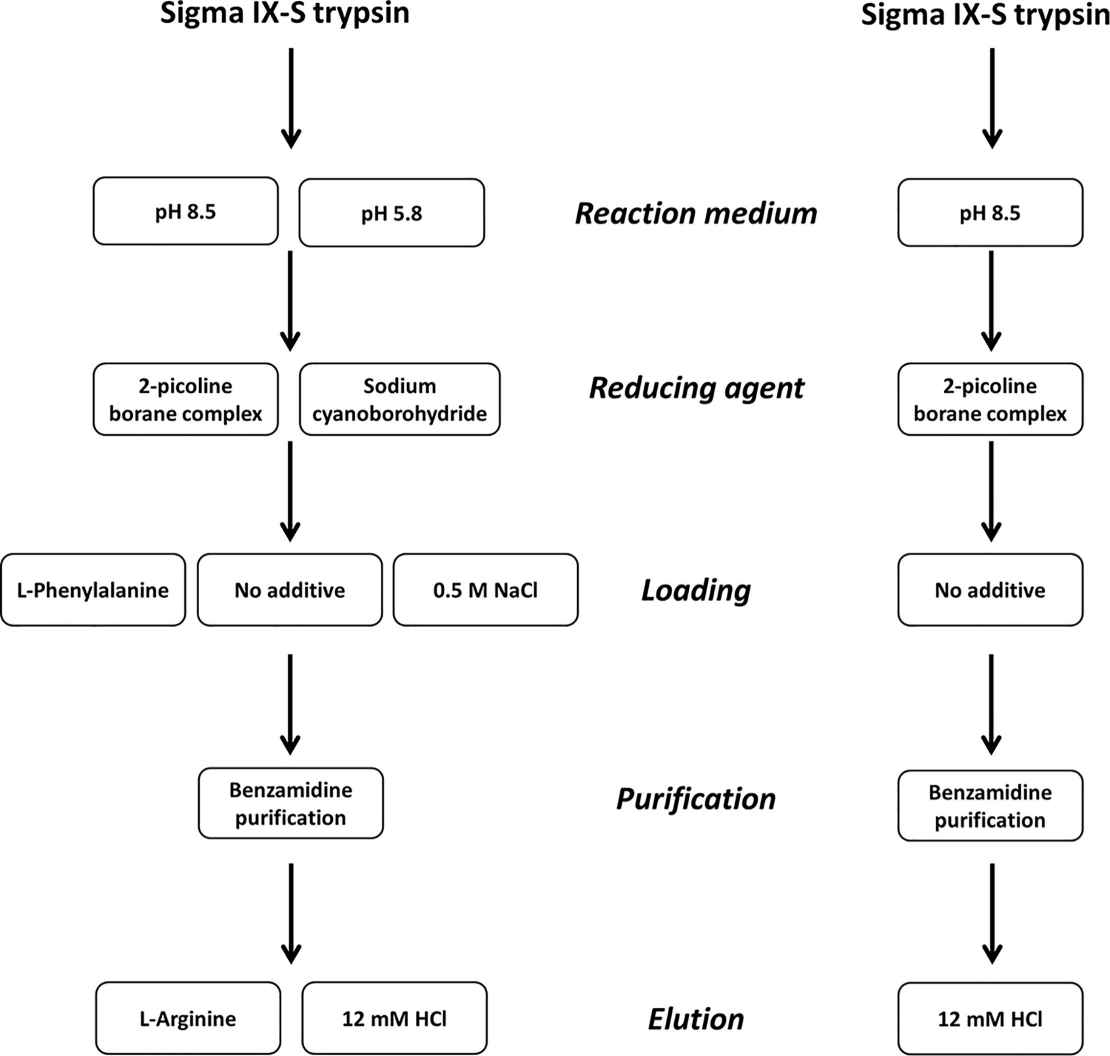
Trypsin preparation workflow. (A) The general workflow for preparation of purified, stabilized trypsin along with different parameters that were evaluated. (B) The final workflow after evaluation.

A flowchart of the protocol is presented in Fig 1, and the complete protocol to the work is presented in S1 Text.

### Amino acid analysis

For several steps in the work, amino acid analysis (AAA) was used to determine the concentration of both enzyme and substrate in addition to monitoring the lysine-to-dimethyllysine conversion[23].

### Reductive methylation

IX-S porcine trypsin (Product number T0303, Sigma-Aldrich, Germany) was solubilized in either 50 mM triethylammonium bicarbonate (TEAB) pH 8.5 or 20 mM NaH_2_PO_4_/Na_2_HPO_4_ pH 5.8 to a final concentration of 1 mg/mL. 2.2 μL 36% formaldehyde per mg trypsin was added followed by the addition of 20 μL 0.6 M NaBH_3_CN in water or 2-picoline borane complex in methanol per mg trypsin. The tube was left at room temperature with rotation for 60 minutes. Aliquots were withdrawn at several time points and purified either by acetone precipitation or reversed-phase purification in order to monitor the lysine-to-dimethyllysine conversion rate. Aliquots were analyzed by both MALDI-TOF and AAA.

### Benzamidine purification

All work in this section was performed at 5°C. One volume (100 μL per mg trypsin) of Fast-Flow Benzamidine-Sepharose 4b beads (GE Healthcase Life sciences, PA, USA) was mixed with three volumes of 50 mM ammonium bicarbonate (AMBIC) pH 8 in a reaction tube of sufficient size for the preparation. The sample was mixed, and the beads were sedimented by brief centrifugation using a hand-powered centrifuge. The supernatant was removed, and a loading buffer was added along with the trypsin solution. The loading buffer consisted of either, 500 mM NaCl, 50 mM AMBIC or saturated L-phenylalanine in 50 mM AMBIC.

The sample was mixed for 30 minutes by rotation, after which the beads were sedimented as described previously. Washing was performed by removing the supernatant and replacing it with 50 mM AMBIC, followed by mixing the beads by rotation for 5 minutes and finally sedimention. The washing step was performed three times in total. Elution was carried out by removing the supernatant and adding three volumes of elution buffer. The elution buffer consisted of either 12 mM HCl pH 1.5 or 1M L-arginine buffered to pH 6. The tube was rotated for 30 minutes followed by sedimentation of the bead. The supernatant was withdrawn and the protein concentration was determined by AAA. The enzymatic assay for trypsin activity was carried out as described in https://www.sigmaaldrich.com/technical-documents/protocols/biology/enzymatic-assay-of-trypsin.html.

### Monitoring autodigestion

The degree of autodigestion was studied by incubating 20 μg of both Sigma IX-S and enhanced trypsin in 50 mM AMBIC (40 ng trypsin/μL) at 37°C and withdrawing aliquots after 1, 2, 3, 4, 6 and 17 hours. The aliquots were acidified by addition of TFA to a final concentration of 1% (v/v). Samples were analyzed by RP-HPLC using a 1260 Infinity HPLC system (Agilent Technologies, Germany) equipped with an Aeris Peptide C18 column (2.1*150 mm, 3.6μm, Phenomenex, CA, USA). Solvent A consisted of 0.1% TFA in water, while solvent B consisted of 0.1% TFA, 80% acetonitrile in water. Solvent B levels were kept at 2% for two minutes, followed by an increase to 10% over eight minutes, followed by a sharp increase to 90% over two minutes, where it was kept for two minutes.

The percentage of intact protein was determined as the peak area of the intact trypsin divided by the total peak area (intact protein plus the resulting peptides) across the run.

### Preparation of simple protein mix

Four standard proteins were selected for evaluating the specificity of trypsin. Bovine serum albumin (product number A7030), horse myoglobin (product number M1882), horse alcohol dehydrogenase (product number A6128), and bovine carbonic anhydrase (product number C3934) were solubilized in 8M urea, 50 mM TEAB, to a final concentration of 3μg/μL. Disulfide bonds were reduced by the addition of dithiothreitol (DTT) to a final concentration of 10 mM and incubated at 30°C for one hour after which iodoacetamide (IAA) was added to a final concentration of 23 mM, and the samples were incubated for 45 minutes in the dark at RT. Excess IAA was quenched by the addition of an extra 2 mM of DTT. The protein solutions were mixed, and 50 mM TEAB was added until a final concentration of 0.8 M urea was reached.

### Preparation of HeLa lysate

A HeLa lysate, which had been purified by ethanol/acetone precipitation, was reduced and alkylated under the same conditions as the simple protein mix. In order to remove urea, which induces carbamylations at high temperatures[24], the sample was purified using an in-house constructed Stage-tip. Micro-quartz fiber filter paper (Ahlstrom Munktell, Sweden) was firmly positioned in the bottom of a D200 pipette tip (Gilson, WI, USA) using a syringe, and a methanolic slurry of Poros R1 20 resin was applied on top. Equilibration and loading was performed using 0.1% TFA in water, and elution was performed in two steps; first using 30% ACN in water followed by 70% ACN in water. The elution was performed directly into a solution of 50 mM AMBIC.

### Digestion conditions

#### Overnight digestion

Sigma trypsin (crude Sigma IX-S, benzamidine-purified Sigma IX-S and enhanced (benzamidine-purified and methylated) Sigma IX-S) and PSG were added at either 2% or 4% w/w to aliquots of the protein mixture, and digestion proceeded at 37°C overnight. The methylated trypsin used in this experiment was subjected to reductive methylation for only 10 minutes. Tryptic activity was stopped by the addition of TFA to a final concentration of 1% v/v, and the samples were placed on ice. A sample clean-up was achieved using home-made micro-purification tips which were constructed by stomping a D10 pipette tip (Gilson, WI, USA) with a C18 Empore disc (3M, MN, USA) and applying a slurry of Poros R2 50 resin in acetonitrile until 2 mg beads dry-weight was placed in the tip. All digestions were performed in duplicates.

#### Digestion at elevated temperatures

Sigma trypsin (crude Sigma IX-S and enhanced Sigma IX-S) and PSG were added at 2% w/w to aliquots of HeLa lysate, which were preheated to 58°C and placed in a heated shaker (HTA-Biotec, Germany). Aliquots were withdrawn at t = 1, 5, 10, 30 and 60 minutes and added directly to wells in a 96-well PCR-plate, which already contained equivolumetric amounts of 10% FA in order to stop the digestion. The samples were lyophilized, redissolved in 0.1% FA, and analyzed by LC-MS. All digestions were performed in duplicates. The reported peptide numbers are average numbers of peptides identified at 1% False Discovery Rate (FDR) between duplicates. For host-cell protein analysis, 1 μg each of non-purified and enhanced Sigma trypsin and PSG were dissolved in 20 μl of 20 mM AMBIC, pH 7.8, and incubated for two hours at 58°C. Samples were reduced, alkylated with IAA, and micro-purified as described for the protein mixture.

### LC-MS/MS analysis

1 μg of each peptide mixture were separated on a 23 minute gradient using an EASY-nLC II (Proxeon, Odense, Denmark) equipped with a 200 mm*75μm ID pulled-emitter column, packed with 3μm Reprosil Pur C18 material (Dr. Maisch, Germany), operating at 250 nL/min. Samples were analyzed using a Q-Exactive HF mass spectrometer (Thermo Scientific, Bremen, Germany). Data was recorded in a Top 10 DDA manner with an MS1 resolution of 120 000 and an MS2 resolution of 15 000.

### Database searching and quantitation

All data was searched using the Andromeda search engine through MaxQuant version 1.5.3.30[25]. All files were searched with default parameters unless otherwise stated. The data was searched by using both non-specific cleavage (for evaluation of specificity) and with trypsin as the protease (for evaluation of activity), with two missed cleavages allowed. The spectra were queried against the sequences of the four standard proteins (retrieved from UniProt) along with common contaminants, including autodigested trypsin. The FDR on both peptide and protein levels was set to 1%. As this is, to a high degree, a peptide-centric study, we report the number of identified peptides at 1% FDR, rather than proteins. We do, however, acknowledge that the 1% protein FDR is an imperfect estimate. When analyzing the HeLa data, the raw files were searched against the Uniprot human proteome (73950 sequences, Feb 12^th^ 2019) along with a custom database consisting of the trypsin contaminants identified in the HCP study. A minimum peptide length of 9 amino acids was required.

An HCP analysis of the various trypsins was performed using the X! Tandem search engine (Alanine) (http://www.thegpm.org). All spectra were queried against the downloaded UniProt Sus scrofa proteome (40.706 sequences, March 10th, 2019) and the most recent cRAP list (http://www.thegpm.org). Data were search as: enzyme: semi-tryptic, parent ion error: 10 ppm, fragment error: 0.1 Da, max e-value: 0.01, fixed modifications: carbamidomethylation (C), partial modifications: oxidation (M), methylation (K), dimethylation (K). At least two peptides were required for a positive identification. Each triplicate set of result files were then analyzed through PeptideShaker 1.16.38 [26] and reported at 1% FDR.

### Data analysis

Data analysis was performed using RStudio version 1.0.136[27]. Tryptic peptides were defined as peptides where both terminals were the result of cleavages C-terminal to lysine or arginine. Chymotryptic peptides were defined as peptides where both terminals resulted from cleavages C-terminal to tryptophan, tyrosine or phenylalanine. Semi-tryptic and semi-chymotryptic peptides were defined as peptides with a specific cleavage at one terminal, while the other terminal was produced by C-terminal cleavage to any other amino acid. Tryptic-chymotryptic peptides were defined as peptides where one terminal was the result of tryptic cleavage, while the other terminal was the result of chymotryptic cleavage. Non-specific peptides were defined as peptides where both terminals were the result of cleavages C-terminal to any other amino acid. If a peptide was located at either the N- or C-terminal of the protein, the given terminal was considered as being tryptic.

## Results and discussion

### Rate of methylation

**Fig 2:**
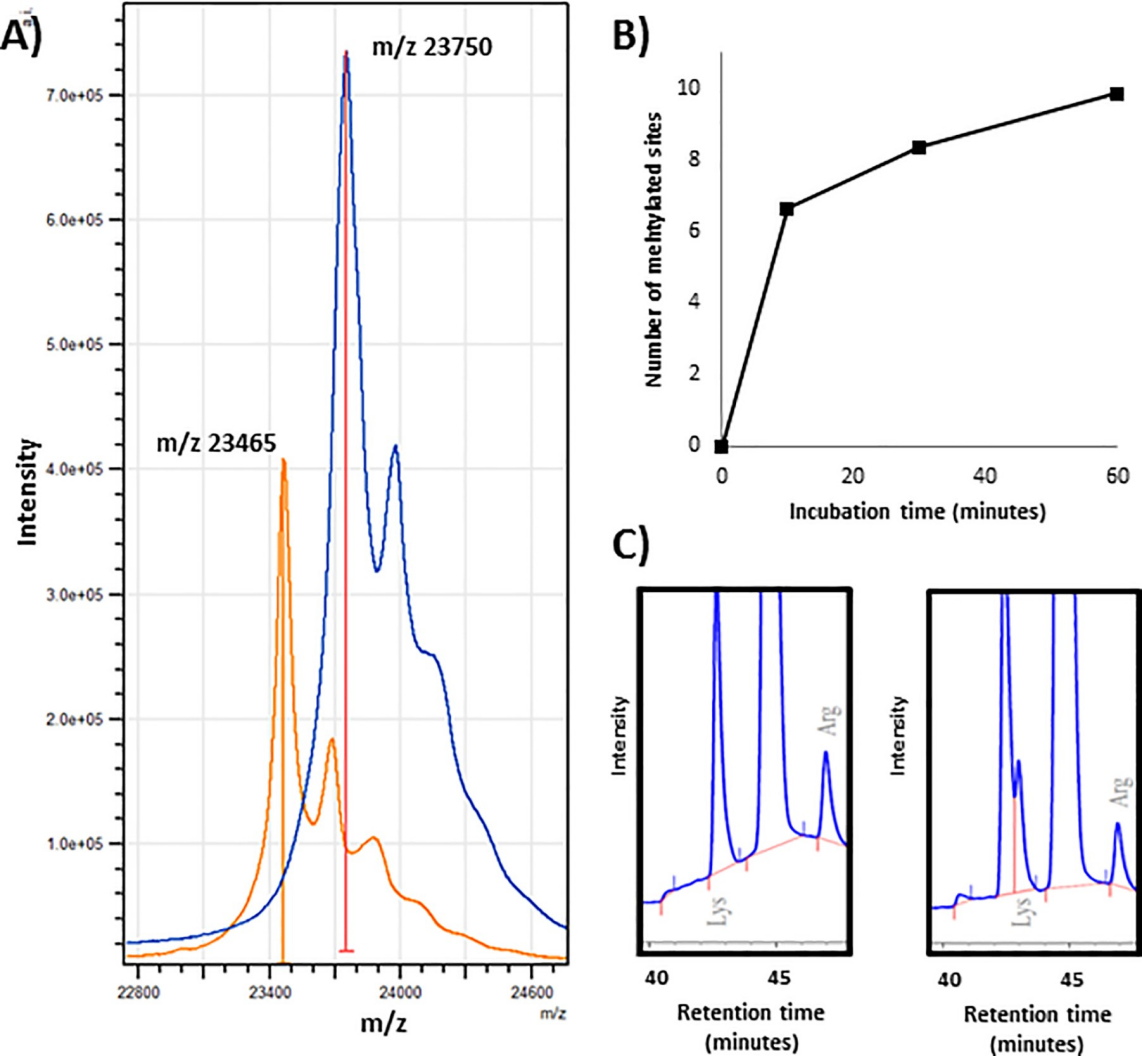
Extent of trypsin methylation. (A) MALDI-TOF spectra of intact Sigma IX-S in the non-methylated (Orange) and 60 min methylated (Blue) form. (B) The lysine content during methylation at pH 5.8 as a function of time measured by AAA. C) AAA chromatograms showing lysine and dimethyllysine in a non-methylated (left) and 60 min methylated (right) sample.

The degree of methylation was determined by MALDI-TOF and AAA. Fig 2A shows examples of MALDI-TOF spectra of non-methylated (top) and methylated (bottom) Sigma IX-S porcine trypsin.

Using TEAB as the reaction medium, the mass of trypsin increased during the first 10 minutes of methylation and did not increase significantly during the remaining time. The AAA results were used to calculate the lysine-to-dimethyllysine conversion, as dimethyllysine was found to produce a separate peak, eluting slightly earlier than lysine (although not fully baseline-separated, see Fig 2C). The peak areas from lysine and dimethyllysine were normalized against the peak area of glycine and the peak ratio at T = 0. After 10 minutes, the lysine content had dropped by 93%.

Using phosphate buffer pH 5.8, the reaction was found to proceed at a significantly lower rate. Fig 2B shows the degree of methylation as a function of time. However, after 60 minutes the same end result was achieved. As the activity of trypsin is much lower at this pH, the level of autodigestion is likely lowered. The reaction medium that produced the lowest amount of autodigestion was not determined.

This information demonstrated that the methylation procedure could be performed much faster than originally expected based on previous protocols, which is advantageous due to the methylation process taking place at conditions where trypsin may perform autodigestion. The average derivatization efficiency was found to be 92% based on the residual lysine content. However, there is a possibility that dimethyllysine degrades during acid hydrolysis, so the degree of methylation may therefore be even higher. No changes in relative amounts were observed for any other amino acid, and no new peaks, except for dimethyllysine, were present in the methylated hydrolysate, when HCl was used for eluting trypsin from the benzamidine beads. When arginine was used as the eluent, dominant arginine and ornithine peaks were observed in the AAA chromatogram along with an unidentified peak eluting between glutamic acid and glycine. Additionally, 2-picoline borane complex was found to be an effective reducing agent and could easily replace sodium cyanoborohydride.

### Benzamidine purification

Various loading and elution strategies were evaluated in order to optimize the yield and specificity of trypsin (see Fig 1 for the overall workflow). Based on AAA data, loading with NaCl significantly decreased the yield of active trypsin from 34.5% to 26.5%. Loading with phenylalanine had no affect on the yield while elution with 1M L-arginine buffered to pH 6 produced a marginally lower yield compared to elution with HCl. The yield from this procedure is reproducible, as shown by three independent purifications in Table 1, each yielding 3.5 mg (35%).

**Table 1:** Purification yields.

	First purification	Second purification
Trypsin 1	3.5 mg	4.0 mg
Trypsin 2	3.5 mg	4.1 mg
Trypsin 3	3.5 mg	2.5 mg

Yields from three individual batches of methylations and purifications of enhanced trypsin as determined by AAA. In order to further reduce the cost of the workflow, the beads from the first round of purification were reused for a second round of purification.

The raw cost of the enhanced trypsin is quite low. The total cost for one batch with a yield of 3.5 mg is around $ 17 (trypsin $ 1.5, chemicals $ 1 and benzamidine beads $ 15). 100 μg of PSG has a listed price of $ 143 while the present procedure yields a price of $ 0.05/100 μg of enhanced trypsin. However, the price does not include infrastructure and the time used for trypsin preparation (approx. four hours for one batch). As the benzamidine beads are the most expensive part of the protocol, we attempted to reuse them for a second round of purification. A more variable yield was observed, as two purification batches showed a slightly higher yield and one batch a lower yield (Table 1). The higher yield is likely due to the fact that not all trypsin elutes in the first round of purification, while the lower yield could be due to loss of benzamidine beads that did not precipitate during the centrifugation steps. Multiple purifications can thus be carried out using the same beads, thereby bringing the price of enhanced trypsin even further down.

### Trypsin activity

The activity of the various trypsin preparations was tested using BAEE (N_α_-Benzoyl-L-arginine ethyl ester) as a substrate. PSG had an activity of 13200 BAEE units, while the activity of the crude Sigma IX-S preparation was 17900 BAEE units. Three independent preparations of enhanced Sigma IX-S were measured at 9100, 9200 and 9400 BAEE units respectively, thus showing a high reproducibility and an activity close to that of PSG.

### Effects of methylation on specificity

**Fig 3:**
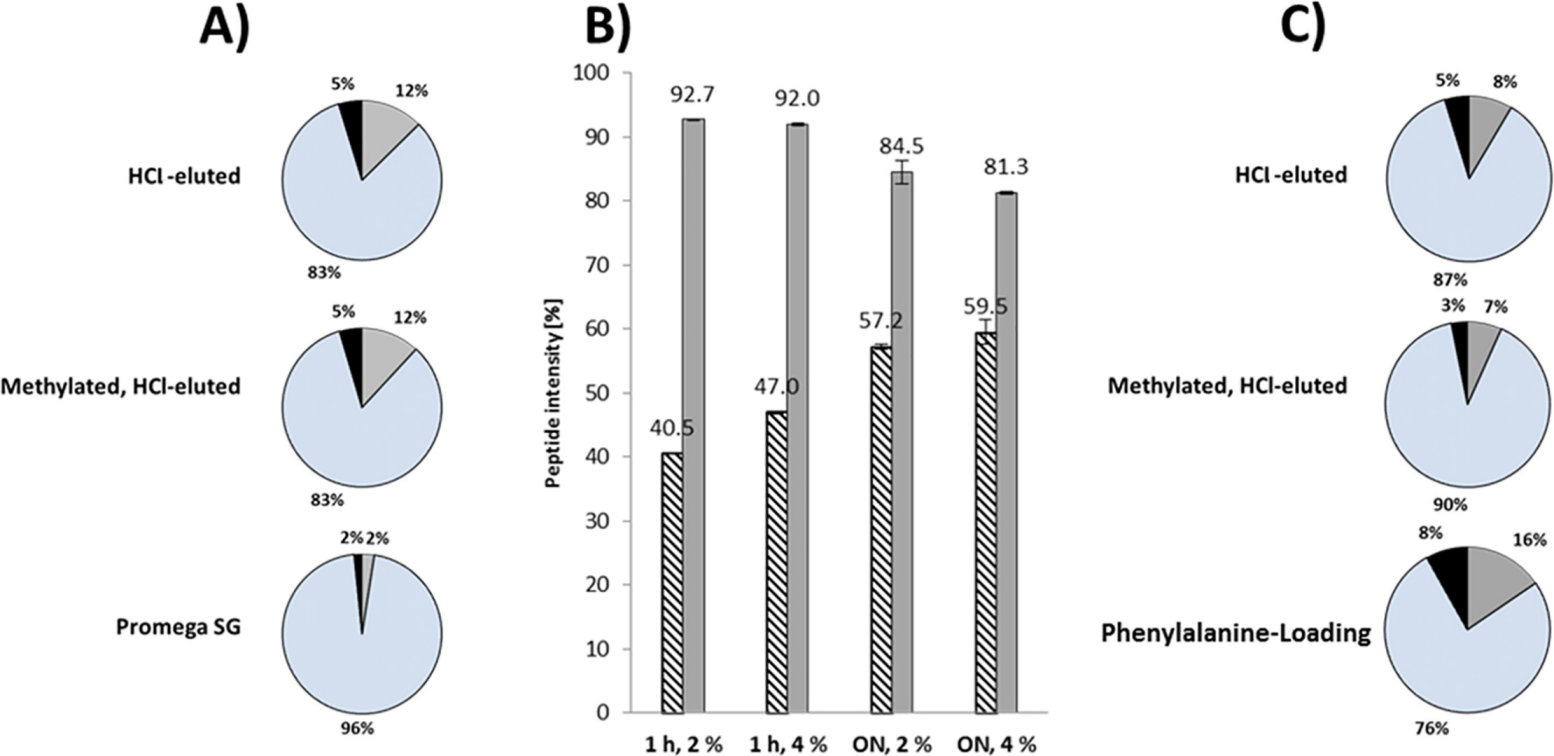
Distribution of tryptic peptides from digests. (A) Distribution of peptide abundances (measured based on peak area) with fully tryptic (blue), tryptic-chymotryptic (gray) and semitryptic (black) peptides for a 37°C overnight digest using different trypsin preparations at 4% trypsin w/w. (B) Percentage of peptide intensities found in a digest of the simple protein mix using enhanced Sigma IX-S trypsin under different incubation times (one hour [1h] or overnight [ON]) and enzyme concentration (2 or 4% w/w trypsin-to-substrate). The plot shows the percentage of peptides with zero missed cleavages (shaded) and peptides with fully tryptic terminals (grey). (C) Distribution of peptide abundances (measured based on peak area) for a 37°C overnight digest using different trypsin preparations at 2% trypsin w/w.

The simple protein mix was digested using the different trypsin preparations and subjected to a non-specific peptide search using the Andromeda search engine in order to determine the cleavage specificities. The distribution of fully tryptic, semitryptic, chymotryptic, semichymotryptic, tryptic-chymotryptic and non-specific peptides was calculated based both on the number of observations and the sum of the peak areas. Although the number of fully tryptic peptides constituted ~50% of all identified peptides for all preparations of Sigma IX-S trypsin and 57% for PSG, the peptide intensities (measured as the peak areas) of the fully tryptic peptides were found to constitute ~83% for all Sigma IX-S preparations and 96% for PSG when digesting with 4% w/w trypsin, as presented in Fig 3A. The remaining peptides were mostly of semitryptic and tryptic-chymotryptic character. These results show that the specificity of Sigma IX-S is not altered by the methylation process. To evaluate the effects of the digestion duration and enzyme concentration, the simple protein mix was digested both for one hour and overnight using both 2% and 4% enhanced Sigma IX-S trypsin-to-protein (w/w). Based on both a specific and a non-specific peptide search, the distribution of fully tryptic peptides, as well as missed cleavages, could be evaluated. As seen from Fig 3B, the combined intensity of fully tryptic peptides with no missed cleavages increased from 40% to ~60% when using overnight digestion and 4% trypsin (w/w) both individually and in combination. However, the combined intensity of the chymotryptic peptides increased with both enzyme concentration and time as shown by a decrease in the intensity of fully tryptic peptides. Chymotryptic cleavage products are primarily present in overnight digestions with 4% trypsin. The trypsin concentration should thus be lowered to 2% for overnight digestions in order to increase the specificity.

When digesting with 2% trypsin, the specificity increased to 87–90% for both crude and enhanced Sigma IX-S trypsin. However, when phenylalanine was used in the loading buffer, the specificity decreased to 76% and chymotryptic activity increased significantly. Fig 3C summarizes the obtained results. No increase in specificity was observed when using arginine as eluent.

Low-abundant cleavage products, such as chymotryptic peptides, are likely to be identified due to the low complexity of the sample, thereby decreasing the apparent specificity of the protease. When analyzing complex samples, the high dynamic range of proteins will mainly lead to identification of the high-abundant species, which means that a higher specificity is expected [27,28].

### Rate of autodigestion

**Fig 4:**
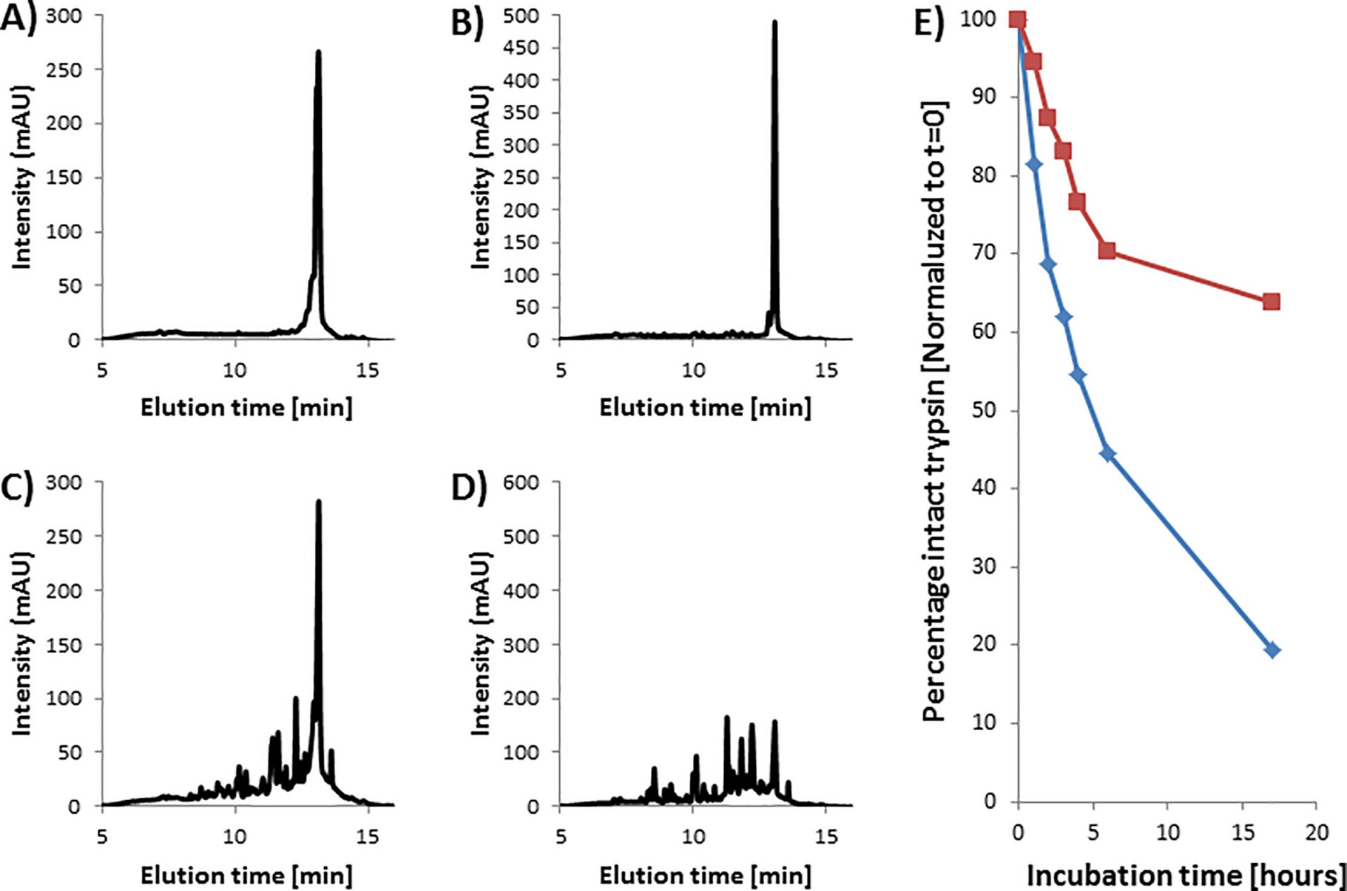
Autodigestion of trypsin. (A)-(D) HPLC chromatograms of trypsin during autodigestion at 37°C. Enhanced trypsin is shown after 0 (A) and 17 (C) hours. Crude Sigma IX-S trypsin is shown after 0 (B) and 17 (D) hours. (E) Stability of crude (blue diamonds) and enhanced (red squares) Sigma-IX-S trypsin towards autodigestion.

The initial HPLC analyses showed that intact trypsin accounted for 76% of the total peak area for the crude Sigma IX-S trypsin, 83% for the benzamidine-purified Sigma IX-S trypsin, and 81% of the enhanced Sigma IX-S trypsin, meaning that autodigest fragments were present to a minor degree even after benzamidine-purification. This is suspected to be a result of residual enzymatic activity from either chymotrypsin or pseudotrypsin, even though the enzymes have been stored in 12 mM HCl. The HPLC analyses determined that the enhanced trypsin performed autodigestion at a much lower rate, and after 17 hours, the ratio of intact trypsin-to-fragments was 64% compared to that at T = 0. For the non-methylated Sigma IX-S trypsin, the ratio had dropped to 20% compared to that at T = 0. This clearly illustrates that methylated trypsin exhibits a higher stability than the non-methylated trypsin, which is in accordance with the literature. Fig 4A and 4D shows HPLC chromatograms of methylated and non-methylated Sigma IX-S trypsin, while Fig 4E shows the rate of autodigestion. These results show that methylation and purification can be used for stabilization of commercial trypsin, which means that trypsin will remain active for longer during the digestion process.

### Host cell proteins analysis

Since contaminating proteins from the trypsin preparations may contaminate the final product, we undertook an analysis of the autodigest products of the various trypsin preparations. All trypsin preparations showed contamination by a number of keratins. As the levels were similar in all preparations and some were grouped as both human and porcine, they are likely to be partly laboratory contaminants. In addition to keratins a number of proteins could be identified at very low levels in all preparations. PSG is clearly the cleanest preparation, with 61 proteins being identified with at least 4 peptides in a triplicate analysis. For crude and enhanced Sigma IX-S trypsin, the 217 and 167 proteins respectively were identified, which shows that the benzamidine purification step did reduce the level of contaminants. For both the crude and enhanced Sigma IX-S trypsin, homologs to chymotrypsin was detected (accession numbers I3LJ52 and I3LHI7), and their levels were not significantly reduced by the purification step. This indicates that the specificity of the preparations may be increased slightly by treating the Sigma IX-S fraction with TPCK[7]. A complete list of identified proteins is presented in S1 Table.

### 1 hour digestions at elevated temperatures

**Fig 5:**
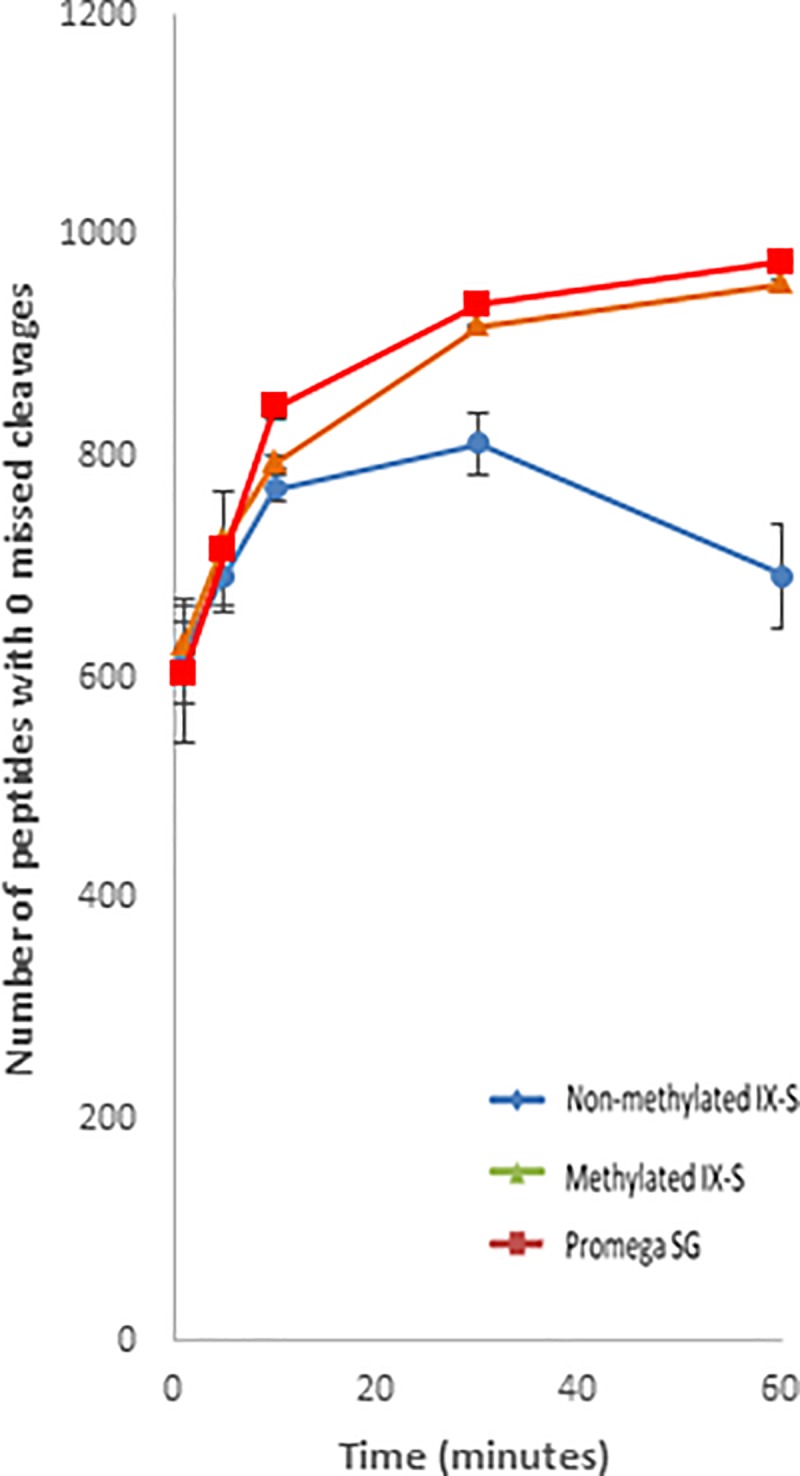
Identified peptides of HeLa lysate. Number of identified peptides with 0 missed cleavages identified after digestion of a HeLa lysate at 58°C using either crude (blue diamonds), enhanced Sigma IX-S (red squares) or PSG (green triangles) trypsin.

One of the major advantages of using enhanced trypsin is the option to perform short time digestions by increasing the temperature. We evaluated the digestion efficiency of the crude and enhanced Sigma IX-S trypsin along with PSG for 60 minute digestions at 58°C. Potential contaminant peptides were not reported. No proteins from the custom HCP database were identified. The results show that the number of peptides with zero missed cleavages as a function of time is very similar for the enhanced Sigma IX-S trypsin (957 identified peptides) and PSG (977 identified peptides). The crude trypsin showed a rapid increase in identified peptides for the first 30 minutes, followed by a drop from 30 to 60 minutes, after which 694 peptides were identified. The number of identified peptides after 60 minutes shows that the two methylated trypsin preparations (PSG and enhanced) provide a superior peptide yield compared to the non-methylated trypsin, which is in accordance with the literature, as presented in Fig 5. The chymotryptic background was furthermore found to be heavily reduced when digesting at 58°C for one hour. The percentage of peptides with fully tryptic terminals was found to increase slightly throughout the digestion, but for all three trypsin preparations, the specificity was found to be between 96.0% and 97.6% (measured as the number of fully tryptic peptides relative to all identified peptides). This reduction in chymotryptic activity may be a result of either heat-induced inactivation of chymotrypsin[29] or a short digestion time, as suggested by the previously obtained results.

## Conclusions

In this study we present a fast and easily replicable workflow for reductive methylation and affinity purification of commercial trypsin for increasing its stability as well as its specificity. The methylation process was found to be complete after 10 minutes at pH 8.5, and the affinity purification could be performed in a few hours. When including the time for determining the concentration, this allows for production of ~3.5 mg trypsin in less than a day, which will sustain most laboratories for several months. The affinity purification served not only to remove reagents from the methylation process, but also to remove autodigestion fragments. The small amount of residual chymotryptic activity will mainly affect the results when analyzing simple protein samples, but will have a limited impact on complex samples and when applying short digestion times at high temperature. For maximum specificity, the amount of trypsin should not be too high–we found that 2% w/w yielded a significantly higher specificity for overnight digestions when compared to 4% w/w. Furthermore, the trypsin aliquots should not be subjected to multiple freeze-thaw cycles.

In-house methylation is a fast and cost-efficient way of increasing the value of cheaper trypsin preparations which may be advantageous for laboratories with a large consumption of trypsin or a limited budget for proteases. This method is fully applicable to TPCK-treated trypsin and other trypsin preparations.

### Further perspectives

We furthermore evaluated *1426-SIGMA* from Sigma-Aldrich, which is a bovine trypsin treated with TPCK. We found that the specificity was marginally higher than Sigma IX-S, but still not quite as high as PSG. A possible strategy to further increase the specificity of trypsin may be to link the N-terminus of a target amino acid to agarose beads, such as NHS-activated agarose. Arginine-agarose could be used as an alternative to benzamidine beads and could lead to selective binding of trypsin[19], which would allow removal of chymotrypsin by discarding the supernatant. If elution from the amino acid bead proved inefficient, phenylalanine-agarose could be used for selective binding of chymotrypsin, and the trypsin-containing supernatant could be transferred to benzamidine beads for further purification. These speculations could be tested if increased specificity is desired.

Even though the sites should be stabilized, cleavage C-terminal to dimethyllysine has been reported[30]. For this reason, it has been proposed that methylated trypsin should be included in the database in order to avoid false positives, to which we highly agree. Another strategy for reducing the number of false positives could be to perform the dimethylation using deuterated formaldehyde, or possibly a 50/50 mixture of CH_2_O/CD_2_O, which would lead to a decrease in the intensity of trypsin-derived peaks.

## Supporting information

S1 Text Protocol for stabilization and purification of trypsin. (DOCX)

S1 Table List of proteins identified in each trypsin preparation: Crude Sigma IX-S, enhanced (methylated) Sigma IX-S and PSG (Promega sequencing grade). (XLSX)

## Abbreviations

AAA: Amino acid analysis
AMBIC: Ammonium bicarbonate
BAEE: N_α_-Benzoyl-L-arginine ethyl ester
FA: Formic acid
LC-MS: Liquid chromatography-mass spectrometry
PSG: Promega sequencing grade trypsin
TEAB: Triethylammonium bicarbonate buffer
TFA: Trifluoroacetic acid
TPCK: Tosyl phenylalanyl chloromethyl ketone

## References

[1] Whitcomb DC, Lowe ME. Human pancreatic digestive enzymes. Dig Dis Sci. 2007;52: 1–17. 10.1007/s10620-006-9589-z 17205399

[2] Keil B. Specificity of Proteolysis. Berlin, Germany: Springer-Verlag; 1992.

[3] Tabb DL, Huang Y, Wysocki V, Yates JR. Influence of Basic Residue Content on Fragmentation Ion Peak Intensities in Low-Energy Collision-Induced Dissociation Spectra of Peptides. Anal Chem. 2004;76: 1243–1248. 10.1021/ac0351163 14987077 PMC2813199

[4] Aebersold R, Mann M. Mass spectometry-based proteomics. Nature. 2003;422: 198–207. 10.1038/nature01511 12634793

[5] Bunkenborg J, Espadas G, Molina H. Cutting edge proteomics: Benchmarking of six commercial trypsins. J Proteome Res. 2013;12: 3631–3641. 10.1021/pr4001465 23819575

[6] Wu F, Zhao M, Zhang Y, Su N, Xiong Z, Xu P. Recombinant acetylated trypsin demonstrates superior stability and higher activity than commercial products in quantitative proteomics studies. Rapid Commun Mass Spectrom. 2016;30: 1059–1066. 10.1002/rcm.7535 27003043

[7] Carpenter FH. Treatment of trypsin with TPCK. Methods in Enzymology. 1967 p. 237 10.1016/S0076-6879(67)11028-8

[8] Keil-Dlouhá V, Zylber N, Imhoff JM, Tong NT, Keil B. Proteolytic activity of pseudotrypsin. FEBS Lett. 1971;16: 291–295. 10.1016/0014-5793(71)80373-3 11945964

[9] Labouesse J, Gervais M. Preparation of chemically defined epsilon N-acetylated trypsin. Eur J Biochem. 1967;2: 215–23. 6078533 10.1111/j.1432-1033.1967.tb00127.x

[10] Sri Ram J, Terminiello L, Bier M, Nord FF. On the mechanism of enzyme action. LVIII. Acetyltrypsin, a stable trypsin derivative. Arch Biochem Biophys. 1954;52: 464–477. 13208272 10.1016/0003-9861(54)90146-0

[11] Nureddin A, Inagami T. Chemical Modification of Amino Groups and Guanidino Groups of Trypsin. Biochem J. 1975;147: 71–81. 10.1042/bj1470071 239704 PMC1165376

[12] Marshall JJ, Rabinowitz ML. Enzyme stabilization by covalent attachment of carbohydrate. Arch Biochem Biophys. 1975;167: 777–779. 10.1016/0003-9861(75)90525-1 1124940

[13] Rice RH, Means GE, Brown WD. Stabilization of bovine trypsin by reductive methylation. Biochim Biophys Acta. 1977;492: 316–321. 10.1016/0005-2795(77)90082-4 560214

[14] Havlis J, Thomas H, Sebela M, Shevchenko A. Fast-response proteomics by accelerated in-gel digestion of proteins. Anal Chem. 2003;75: 1300–1306. 10.1021/ac026136s 12659189

[15] Šebela M, Štosová T, Havliš J, Wielsch N, Thomas H, Zdráhal Z, et al Thermostable trypsin conjugates for high-throughput proteomics: Synthesis and performance evaluation. Proteomics. 2006;6: 2959–2963. 10.1002/pmic.200500576 16637014

[16] Capelo J et. a. Overview on modern approaches to speed up protein identification workflows relying on enzymatic cleavage and mass spectrometry-based techniques. Anal Chim Acta. 2009;650: 151–159. 10.1016/j.aca.2009.07.034 19720186

[17] Sato S, Sakamoto T, Miyazawa E, Kikugawa Y. One-pot reductive amination of aldehydes and ketones with α-picoline-borane in methanol, in water, and in neat conditions. Tetrahedron. 2004;60: 7899–7906. 10.1016/j.tet.2004.06.045

[18] Wilson-Grady JT, Haas W, Gygi SP. Quantitative comparison of the fasted and re-fed mouse liver phosphoproteomes using lower pH reductive dimethylation. Methods. Elsevier Inc.; 2013;61: 277–286. 10.1016/j.ymeth.2013.03.031 23567750

[19] Chamrad I, Strouhal O, Rehulka P, Lenobel R, Sebela M. Microscale affinity purification of trypsin reduces background peptides in matrix-assisted laser desorption/ionization mass spectrometry of protein digests. J Proteomics. 2011;74: 948–957. 10.1016/j.jprot.2011.02.011 21345391

[20] Smith RL, Shaw E. Pseudotrypsin—A modified bovine trypsin produced by limited autodigestion. J Biol Chem. 1969;244: 4704–4712. 5817636

[21] Mitterer A, Tauer C, Reiter M, Mundt W. Method of isolation and purification of trypsin from pronase and use thereof. EP1456228 B1, 2011. p. EP1456228 B1.

[22] Vizcaíno JA, Csordas A, Del-Toro N, Dianes JA, Griss J, Lavidas I, et al 2016 update of the PRIDE database and its related tools. Nucleic Acids Res. 2016;44: D447–D456. 10.1093/nar/gkv1145 26527722 PMC4702828

[23] Højrup P. Analysis of Peptides and Conjugates by Amino Acid Analysis. Methods in Molecular Biology. Springer; 2015 pp. 65–76. 10.1007/978-1-4939-2999-3 26424264

[24] Stark GR, Stein WH, Moore S. Reactions of the Cyanate Present in Aqueous with Amino Acids and Proteins. J Biol Chem. 1960;235: 3177–3181.

[25] Cox J, Mann M. MaxQuant enables high peptide identification rates, individualized p.p.b.-range mass accuracies and proteome-wide protein quantification. Nat Biotechnol. 2008;26: 1367–1372. 10.1038/nbt.1511 19029910

[26] R Core Team. R: A language and environment for statistical computing. Vienna, Austria: R Core Team; 2016.

[27] Picotti P, Aebersold R, Domon B. The implications of proteolytic background for shotgun proteomics. Mol Cell Proteomics. 2007;6: 1589–1598. 10.1074/mcp.M700029-MCP200 17533221

[28] Olsen J V, Ong S-E, Mann M. Trypsin Cleaves Exclusively C-terminal to Arginine and Lysine Residues. Mol Cell Proteomics. 2004;3: 608–614. 10.1074/mcp.T400003-MCP200 15034119

[29] Ásgeirsson B, Bjarnason JB. Structural and kinetic properties of chymotrypsin from atlantic cod (Gadus morhua). Comparison with bovine chymotrypsin. Comp Biochem Physiol—Part B. 1991;99: 327–335. 10.1016/0305-0491(91)90050-N 1764912

[30] Schittmayer M, Fritz K, Liesinger L, Griss J, Birner-Gruenberger R. Cleaning out the Litterbox of Proteomic Scientists Favorite Pet: Optimized Data Analysis Avoiding Trypsin Artifacts. J Proteome Res. 2016;15: 1222–1229. 10.1021/acs.jproteome.5b01105 26938934 PMC4820788

