# Supplementary material for: Enhanced trypsin on a budget: Stabilization, purification and high-temperature application of inexpensive commercial trypsin for proteomics applications
Source: PLoS One. 2019 Jun 27;14(6):e0218374. doi: 10.1371/journal.pone.0218374 (PMC6597055; doi:10.1371/journal.pone.0218374)
Supplement: S1 Text — (DOCX) [file pone.0218374.s001.docx]

# Purpose

This protocol describes how to increase stability and specificity of cheaper trypsin preparations. For the work carried out in the study, we used IX-S porcine trypsin from Sigma-Aldrich. This protocol is designed for 10 mg trypsin, which will yield ~4 mg after purification.

# Reagents

- 10 mg IX-S trypsin (Sigma Aldrich)
- 50 mM TEAB in H_2_O (500 µL per mg of trypsin)
- 0.6 M 2-picoline borane complex in methanol (prepare fresh)
- 4 % formaldehyde in H_2_O (prepare fresh)
- Benzamidine-sephararose beads (BS4 )
- 50 mM ammonium bicarbonate (AMBIC) in H_2_O
- 10 mM ammonium bicarbonate (AMBIC) in H_2_O
- 1 M AMBIC in H_2_O
- 12 mM HCl in H_2_O

# Procedure

## Preparation

- Take out BS4 and shake it well.
- In a 15 mL reaction tube, mix 1.0 mL BS4 and 3.0 mL 50 mM AMBIC and allow it to settle.
- Discard supernatant.

## Reductive methylation of trypsin

1. Weigh out 10 mg trypsin and place on ice until needed.
2. Dissolve trypsin in 5 mL 50 mM TEAB
3. Add 200 µL 4 % formaldehyde and 200 µL 0.6M 2-picoline borane complex to the sample.
4. Mix carefully.
5. Incubate at RT on a rotator under gentle rotation for 10 minutes.
6. Add 1 mL 1M AMBIC to quench the reaction, and rotate for 5 minutes.

## Purification of trypsin (Should be performed in cold-room)

1. Add the BS4 solution to the trypsin solution.
2. Rotate gently for 30 min and allow the beads to settle by brief centrifugation using a hand-powered centrifuge.
3. Discard supernatant and keep the beads.
4. Wash with 5 mL 50 mM AMBIC.
5. Rotate for 5 min, allow it to settle and remove the supernatant.
6. Repeat step 4 and 5.
7. Remove supernatant and replace with 5 mL 10 mM AMBIC
8. Rotate for 5 min, allow it to settle and remove the supernatant (It is important that the supernatant is efficiently removed).
9. Add 5 mL 12 mM HCl.
10. Rotate gently for 30 min and allow the beads to settle.
11. Withdraw **and keep** the supernatant.
12. Aliquot the trypsin-containing supernatant and freeze at -18°C.

## Verification

1. Withdraw 1 µL of trypsin and determine exact mass using MALDI-TOF or similar method. Include non-methylated trypsin for exact mass-shift.
2. Take 15 µL and analyze by AAA. This will give the exact concentration along with the lysine-dimethyllysine relationship.

# Critical steps

1. After dissolving trypsin, do not leave it for extended time before adding the dimethylation reagents, or it will perform autolysis.
2. When removing the AMBIC solution immediately before adding the eluent, beware to remove as much supernatant as possible, as it otherwise may affect the pH or arginine concentration.

# Anticipated results

The procedure will provide a highly stable trypsin which can be used either at 37°C or at elevated temperatures for shorter digestion periods. The yield may vary, but was around 40 % in our analyses.

# Comments

If a hand-powered centrifuge is not available, the beads can be sedimented by placing the tubes in an upright position for 15 minutes. This, however, increases the sample-handling time significantly.
